# Identifying and exploiting combinatorial synthetic lethality by characterizing adaptive kinome rewiring of EGFRvIII-driven glioblastoma

**DOI:** 10.1186/s40478-025-02068-y

**Published:** 2025-06-28

**Authors:** Benjamin Lin, Abigail K. Shelton, Erin Smithberger, Julia Ziebro, Kasey R. Skinner, Ryan E. Bash, Richard Kirkman, Allie Stamper, Madison Butler, Alex Flores, Steven P. Angus, Michael P. East, Timothy F. Cloughesy, David A. Nathanson, Michael E. Berens, Jann N. Sarkaria, Zev A. Binder, Donald M. O’Rourke, Timothy C. Howton, Brittany N. Lasseigne, Christopher D. Willey, Gary L. Johnson, Anita B. Hjelmeland, Frank B. Furnari, C. Ryan Miller

**Affiliations:** 1 Medical Scientist Training Program, Heersink School of Medicine, University of Alabama at Birmingham, Birmingham, AL USA; 2 Division of Neuropathology, Department of Pathology, Heersink School of Medicine, University of Alabama at Birmingham, WTI 410C, 1824 6th Avenue South, Birmingham, AL 35294-3300 USA; 3 Department of Biology, University of North Carolina, Chapel Hill, NC USA; 4 University of North Carolina School of Medicine, Chapel Hill, NC USA; 5 Department of Pediatrics, Indiana University School of Medicine, Indianapolis, IN USA; 6 Department of Pharmacology, University of North Carolina School of Medicine, Chapel Hill, NC USA; 7 Department of Neurology, David Geffen School of Medicine, University of California, Los Angeles, Los Angeles, CA USA; 8 Department of Molecular and Medical Pharmacology, David Geffen School of Medicine, University of California, Los Angeles, Los Angeles, CA USA; 9 Cancer and Cell Biology Division, Translational Genomics Research Institute, Phoenix, AZ USA; 10 Department of Radiation Oncology, Mayo Clinic, Rochester, MN USA; 11 Department of Neurosurgery and Glioblastoma Translational Center of Excellence, Abramson Cancer Center, Perelman School of Medicine, University of Pennsylvania, Philadelphia, PA USA; 12 Department of Cell, Developmental and Integrative Biology, Heersink School of Medicine, University of Alabama at Birmingham, Birmingham, AL USA; 13 O’Neal Comprehensive Cancer Center, Heersink School of Medicine, University of Alabama at Birmingham, Birmingham, AL USA; 14 Comprehensive Neuroscience Center, Heersink School of Medicine, University of Alabama at Birmingham, Birmingham, AL USA; 15 Department of Radiation Oncology, University of Alabama at Birmingham, Birmingham, AL USA; 16 Department of Medicine, Division of Regenerative Medicine, University of California, San Diego, San Diego, CA USA

**Keywords:** EGFR, Glioblastoma, Synthetic lethality, Kin­ome rewiring, Upfront combinatorial therapy

## Abstract

GBM is an aggressive primary malignant brain tumor that has a poor prognosis. Molecular characterization of GBM has shown that EGFR mutations are present in over 50% of tumors. However, EGFR inhibitors have not shown clinical efficacy in contrast to other EGFR-driven neoplasms due to the unique EGFR biology found in GBM. Upfront combinatorial therapy featuring EGFR tyrosine kinase inhibitors (TKI) may overcome these challenges. To identify combinatorial drug targets within the kinome, we temporally characterized drug-induced kinome rewiring in isogenic, genetically engineered Cdkn2a-deleted mouse astrocytes expressing human EGFRvIII. We utilize RNA sequencing and multiplex inhibitor beads, coupled with mass spectrometry, to demonstrate that kinome rewiring exhibits both shared and unique kinases after acquired resistance develops to EGFR TKI, despite using models with a common genetic background. Additionally, we noted that kinases altered in the acute setting are distinct from those in acquired resistance. By identifying kinome vulnerabilities throughout the acute, dynamic drug response process, we generated a kinase signature associated with EGFR inhibition. Further molecular interrogation of signature genes revealed that drug treatment induces an unexpected increase in Cdk6 protein, but not mRNA, despite live cell imaging and transcriptomic evidence indicating decreased proliferation. Survival experiments with orthotopic allografts show that upfront combination inhibition of Cdk6, using abemaciclib, and EGFR, using neratinib, significantly prolonged median survival compared to neratinib alone. Our findings suggest that identifying and inhibiting targets with synthetic lethality in the upfront combinatorial setting is a viable approach for precision oncology and may help provide an avenue to overcome the resistance mechanisms that contributed to the failures of EGFR as a molecular target in GBM.

**Supplementary Information:**

The online version contains supplementary material available at 10.1186/s40478-025-02068-y.

## Introduction

Glioblastoma (GBM) is the most common malignant primary brain tumor, with a dismal median overall survival of 15 months from initial diagnosis. Current therapeutic strategies, including surgical resection, radiation therapy, and chemotherapy, have been unable to prevent recurrence [45]. GBM has been genomically characterized through multiple large-scale scientific collaborations, including The Cancer Genome Atlas (TCGA) [8, 48]. These studies identified several reproducible transcriptional subtypes, driven primarily by mutated oncogenes, with all subtypes present in a single patient’s tumor [42, 43]. Despite these scientific advances, there is no precision oncology approach for treating GBM that prolongs overall survival. EGFR mutations, which define the classical transcriptional subtype, are present in over 50% of GBMs and drive gliomagenesis, making mutant EGFR a logical target for personalized medicine approaches [8].

The most common EGFR mutation in GBM is an in-frame, interstitial deletion of exons 2–7, known as EGFRvIII [5, 24, 28]. EGFRvIII encodes a truncated, ligand-independent, constitutively active receptor that drives tumor growth and proliferation through three main canonical signaling pathways: MAPK, JAK/STAT, and PI3K [17, 24, 33]. Blockade of EGFR signaling through small-molecule tyrosine kinase inhibitors (TKI) or targeted biologics has thus far been unsuccessful in GBM treatment, which contrasts with other EGFR-driven neoplasms, such as non-small cell lung cancer (NSCLC) [50]. EGFR biology in GBM is complex, and GBM cells adapt to EGFR inhibition to maintain therapeutic resistance [24]. Gatekeeper EGFR mutations, which enable inherent TKI resistance (e.g., T790M in NSCLC), have not been identified in GBM [52]. Instead, GBM adapts to EGFR inhibition through acquired resistance mechanisms, including adaptive bypass signaling, extrachromosomal DNA, and paracrine inflammatory signaling [30, 35, 53].

Due to these challenges, a subset of the neuro-oncology community has discarded EGFR as a molecular target for GBM. However, we can effectively target it through upfront combinatorial therapy that exploits synthetic lethality. Several other cancers have demonstrated that this approach can resurrect molecular targets that were abandoned due to an initial lack of clinical efficacy as single agents. Indeed, BRAF inhibitors in colorectal cancer, CDK4/6 inhibitors in breast cancer, and MEK inhibitors in melanoma all initially failed as single agents but are currently used in upfront combinations in their respective disease settings [18, 26, 34, 36]. Identifying combinatorial agents that target EGFR-driven GBM is a significant unmet need.

Understanding the role of kinome rewiring in acquired resistance to TKIs is crucial for developing effective EGFR-directed precision therapeutics for GBM. The kinome comprises 538 enzymes, ranging from receptors to signaling intermediates, which cooperate to drive dynamic cellular processes. Previous studies have shown that targeting EGFR signaling results in acute upregulation of alternative receptor tyrosine kinases (RTK) in GBM, such as ERBB2, MET, and PDGFRβ [2, 19, 24]. However, the downstream effects of acquired resistance on the GBM kinase transcriptome and proteome have not been thoroughly examined, particularly in the context of EGFRvIII-driven disease. While previous studies have shown upregulation of specific RTK after EGFR inhibition, examination of pan-kinome reprogramming during the acute phase and upon acquired resistance has not been investigated. Drug-induced kinome rewiring may be a contributing factor to the early failures of targeted EGFR therapeutics in GBM clinical trials. We therefore hypothesized that a proteotranscriptomic examination of EGFR TKI-induced kinome rewiring would enable the exploitation of combinatorial therapies, given that over 80 kinase inhibitors have received FDA approval [39].

Here, we used an isogenic preclinical model of EGFRvIII-driven GBM to characterize drug-induced kinome rewiring temporally [51]. We first established baseline signatures using cell lines that have acquired resistance to EGFR TKI. We then describe the dynamics of acute EGFR TKI-induced kinome rewiring over 48 h. Using the kinase signatures identified from this temporal characterization, we identified candidate druggable kinases for upfront combinatorial therapy. Both unique and shared kinases participated in kinome rewiring in resistant cell lines. However, kinases altered during acute kinome rewiring were distinct from those identified after the acquisition of resistance. Our combined proteotranscriptomic characterization of acute kinome rewiring suggested Cdk6 as a candidate secondary target. Orthotopic allografts treated with an EGFR TKI and a Cdk6 inhibitor showed significantly increased survival compared to those treated with EGFR TKI alone. Our study provides a framework for examining the acute, dynamic response of the kinome to identify exploitable therapeutic vulnerabilities that can extend survival in the upfront combinatorial therapy setting.

## Materials and methods

### Cells

CEv3, E4, E5, G1, G5, G8, and G12 cells acquired resistance to EGFR TKI were generated and molecularly characterized as described [51]. E4, E5, G1, G5, G8, and G12 have acquired resistance to EGFR TKI [51]. Briefly, primary astrocytes were isolated from Cdkn2a^−/−^ mice and transduced with retrovirus encoding human EGFRvIII [4]. Resistant cell lines were generated through serial passage in increasing concentrations of the first-generation EGFR TKI erlotinib or gefitinib [51]. Cell lines were maintained as adherent cultures at 37° C and 5% CO2 in Dulbecco’s Modified Eagle Medium (DMEM) supplemented with either 10% (full serum, FS) or 0.5% (starved serum, SS) fetal bovine serum (FBS). Resistant lines were maintained in media containing either erlotinib or gefitinib at 2 µg/ml.

### RNA sequencing (RNA-seq)

RNA was extracted using a RNeasy kit (Qiagen, Hilden, Germany). RNA libraries were prepared using KAPA Stranded RNA-seq kits (Kapa Biosystems, Wilmington, MA) according to the manufacturer’s protocol. RNA libraries were randomized into pools of 12 samples and diluted to 1.65 pM immediately before sequencing. High-throughput sequencing of 75-base-pair single-end reads was performed using a NextSeq 500/550 High Output 75-cycle kit on a NextSeq 500 or NextSeq 550 (Illumina, San Diego, CA). Subsequent data processing and analysis of FASTQ files were performed on a high-performance cluster (HPC), and the data were analyzed using R.

### RNA-seq data preprocessing and analysis

Reads were aligned with STAR to a custom genome derived from Gencode vM32 and vH43 EGFR (hEGFR) [11]. Bam files were indexed with samtools [10, 21]. Aligned transcripts were quantified using Salmon [37]. Salmon quantification files were imported into R using the tximport package. Non-protein-coding genes and genes with low read counts were filtered out. Differentially expressed (DE) genes were identified using the R package DESeq2 as having an absolute log2 fold change (L2FC) > 1 and a false discovery rate (FDR) < 0.05 [27].

### Multiplexed inhibitor bead mass spectroscopy (MIB-MS)

Dynamic kinome profiling was performed using multiplexed inhibitor beads coupled with mass spectrometry (MIB-MS), as we described [12, 30]. Briefly, cell lysates were passed over columns of type I kinase inhibitors (VI-16832, CTx-0294885, PP58, Purvalanol B, BKM-120, UNC-2147 A, and UNC-8088 A) immobilized on Sepharose beads by gravity flow. Bound kinases were eluted, and the buffer was exchanged via chloroform/methanol extraction before overnight digestion with trypsin. The resulting peptides were desalted with PepClean C18 Spin Columns (Thermo Scientific). For dynamic time-course proteomics experiments, peptides were labeled with tandem mass tags (TMT) before desalting. For all other experiments, label-free quantification (LFQ) was used. Peptides were separated over a 120-minute method for LC/MS/MS consisting of a 95-minute linear gradient from 5 − 30% followed by a 12-minute linear gradient from 30 − 45%, 1 min from 45 to 100%, and 12 min at 100% acetonitrile on an EASY-nLC coupled with a QExactive HF mass spectrometer (Thermo Scientific). The mass spectrometer was operated in data-dependent acquisition mode, sequencing the top 15 precursor ions with the following settings for MS1: resolution, 120,000; scan range, 350–1600 m/z; AGC target, 3 × 10^6^; maximum injection time, 100 ms. MS2 settings were as follows: resolution, 15,000; scan range, 200–2000 m/z; AGC target, 1 × 10^5^; and maximum injection time, 60 ms. The resulting spectra were mapped to the mouse SwissProt proteome, supplemented with the human EGFR protein sequence, and quantified using MaxQuant and the integrated Andromeda search engine. Default settings with match between runs were enabled in MaxQuant, with one exception: the matching time window was set to 4 min. Fixed modifications included carbamidomethyl cysteine, and variable modifications included methionine oxidation and protein N-terminal acetylation. Subsequent data processing and analyses were performed in R.

### MIB-MS data analysis

Raw protein group intensities from MaxQuant output files were imported into R and filtered to include only kinases. Missing values were imputed using the DEP package with the following settings: fun = “man,” shift = 3, and scale = 0.3 [54]. Differentially expressed genes were identified using the R package limma with an FDR < 0.05 [38]. TMT labeling experiments with multiplexed samples used the relative abundance of the respective isobaric tag from MaxQuant output files.

### Downstream data analysis

Raw RNA-seq counts and log2-transformed protein intensities were used for differential expression analysis, with batch effects incorporated into the design formula. Raw RNA-seq counts were transformed using a variance-stabilizing transformation or DESeq2 normalization for visualization. Protein group intensities were transformed using the limma removeBatchEffects function for visualization. Volcano plots were created in R from DESeq2 or limma outputs using EnhancedVolcano [6]. Hierarchical clustering heatmaps were generated in R from transformed mRNA read counts or protein group intensities using the ComplexHeatmap package with center scaling for visualization [15, 16]. mRNA or protein counts were extracted by sample and time point, and the median counts were determined for each group. Z-scores were calculated for each gene across the four time points. Z scores were scaled across the captured transcriptome or kinome. After filtering for DE genes, K-means clustering was performed using 2 clusters. If the mean difference between the 48-hour timepoint and the 0-hour timepoint was positive, then the cluster was labeled “up,” and if the mean difference was negative, the cluster was labeled “down”. Genes were labeled as differentially expressed (DE) if any pairwise comparisons with the 0-hour baseline were identified by DESeq2 or limma. Functional enrichment analysis was performed using gProfiler2 in R. Mouse gene symbols were converted to human gene symbols. Gene sets from Gene Ontology: Biological Processes were used for enrichment analysis. Protein-Protein interaction networking was performed using STRING-DB (https://string-db.org/) [41]. Downstream data analysis was performed using a publicly available Docker container (https://github.com/benlin-UAB/240306_Docker_R-4.3.2 ) on Docker Hub (https://hub.docker.com/repository/docker/benjaminlin1/240306_rstudio_4.3.2) [22]. Analysis was performed on a locally hosted instance of RStudio Server running R version 4.3.2 on the x86_64-pc-linux-gnu (64-bit) platform and the Ubuntu 22.04.4 LTS operating system within a Docker container. Refer to the GitHub repository (https://github.com/benlin-UAB/2025_Lin_ComboSyntheticLethality_Manuscript) for a comprehensive list of installed packages and their versions [23].

### Baseline kinase resistance signatures

Each resistant cell line was compared to CEv3 cells across culture conditions. DE kinase proteins were identified using limma [38]. DE kinases were categorized as either shared among the comparisons or unique to a single cell line and then visualized using upset plots from ComplexHeatmap [15, 16]. hEGFR and ambiguous DE kinases unique to one culture condition and shared in another were removed to simplify the analysis.

### EGFR inhibitor (EGFRi) signature

DE kinases from CEv3, E4, E5, G1, G5, G8, and G12 cells were identified after treatment with afatinib for 4, 24, and 48 h compared to cells not receiving the drug. DE kinases for each cell line were labeled as “up” or “down” by K-means clustering. DE kinases that had ambiguous labeling, labeled as “up” in one cell line and “down” in another cell line or vice versa, were removed. DE kinases for the CEv3 cell line were compared with DE kinases in all resistant cell lines. DE kinases that were unique to CEv3 cells after afatinib treatment were defined as the EGFRi signature.

### Drugs and dose-response studies

Afatinib, neratinib, and abemaciclib were purchased from MedChem Express (Monmouth Junction, NJ) and dissolved in dimethyl sulfoxide (DMSO). To determine drug sensitivity, cells were plated in 96-well plates with at least five technical replicates per cell line. The next day, cells were treated with DMSO or drug. The effects of drugs on cell growth were assessed 72 h after treatment using phase-contrast live cell imaging with a Cytation 5 imager and Gen5 software (Agilent, Santa Clara, CA). Cell counts were fit to a non-linear log [inhibitor] versus response curve with variable slope, and IC_50_ values were calculated using GraphPad Prism (La Jolla, CA). To determine the effects of drugs on proliferation in real time, CEv3 cells were plated in 96-well plates with at least four technical replicates. The next day, cells were treated with DMSO or the drug and counted every 2 h using live-cell imaging with the Incucyte live-cell imaging system (Sartorius, Germany). Live cell counts were averaged among the technical replicates and plotted in GraphPad Prism.

### Orthotopic allografts

These studies were conducted in accordance with the policies of the University of Alabama at Birmingham Institutional Animal Care and Use Committee (IACUC). CEv3 cells were harvested and orthotopically injected as described [31]. For each treatment arm, 100,000 CEv3 cells were orthotopically injected into 10 mice. Treatment commenced 7 days later. Neratinib was suspended in Cremophor and administered by oral gavage daily, q5, at 50 mg/kg. Abemaciclib was suspended in saline and administered by oral gavage daily, q5, at 50 mg/kg. Saline was used for the control arms.

## Results

### CEv3 cells provide an isogenic modeling platform to study drug-induced kinome rewiring in EGFRvIII-driven GBM

EGFR mutations drive tumorigenesis in over 50% of GBM [8]. The EGFRvIII variant is an interstitial deletion mutant that encodes a ligand-independent, constitutively active kinase, driving gliomagenesis [33]. Despite clinical success in other neoplasms, targeting EGFR has not yielded significant benefits for patients with GBM. To further understand these failures, we sought to characterize kinome rewiring in isogenic, genetically engineered mouse astrocyte models. CEv3 cells (Fig. S1A) were generated from parental astrocytes (C cells) isolated from a genetically engineered mouse harboring homozygous Cdkn2a deletion, a mutation that frequently co-occurs in GBM. CEv3 cells express human EGFRvIII (hEGFR) and provide an isogenic EGFRvIII-driven model well-suited for this study. CEv3 cells rapidly form tumors when orthotopically implanted in mice [51]. To determine how EGFRvIII expression programs the kinomic landscape, we compared RNA-seq and proteomics data from CEv3 cells with those of control cells (C) cultured in the absence of drug. Kinases are typically expressed at relatively low levels, limiting the capacity of standard mass spectrometry workflows to quantify a large percentage of the expressed kinome. For our proteomics studies, we employed affinity capture of functional kinases based on their ability to bind Type I kinase inhibitors as ATP analogs, using multiplexed inhibitor beads (MIB) [9]. Subsequent mass spectrometry (MS) routinely quantified approximately 300 kinases in a single MS run.

**Fig. 1:**
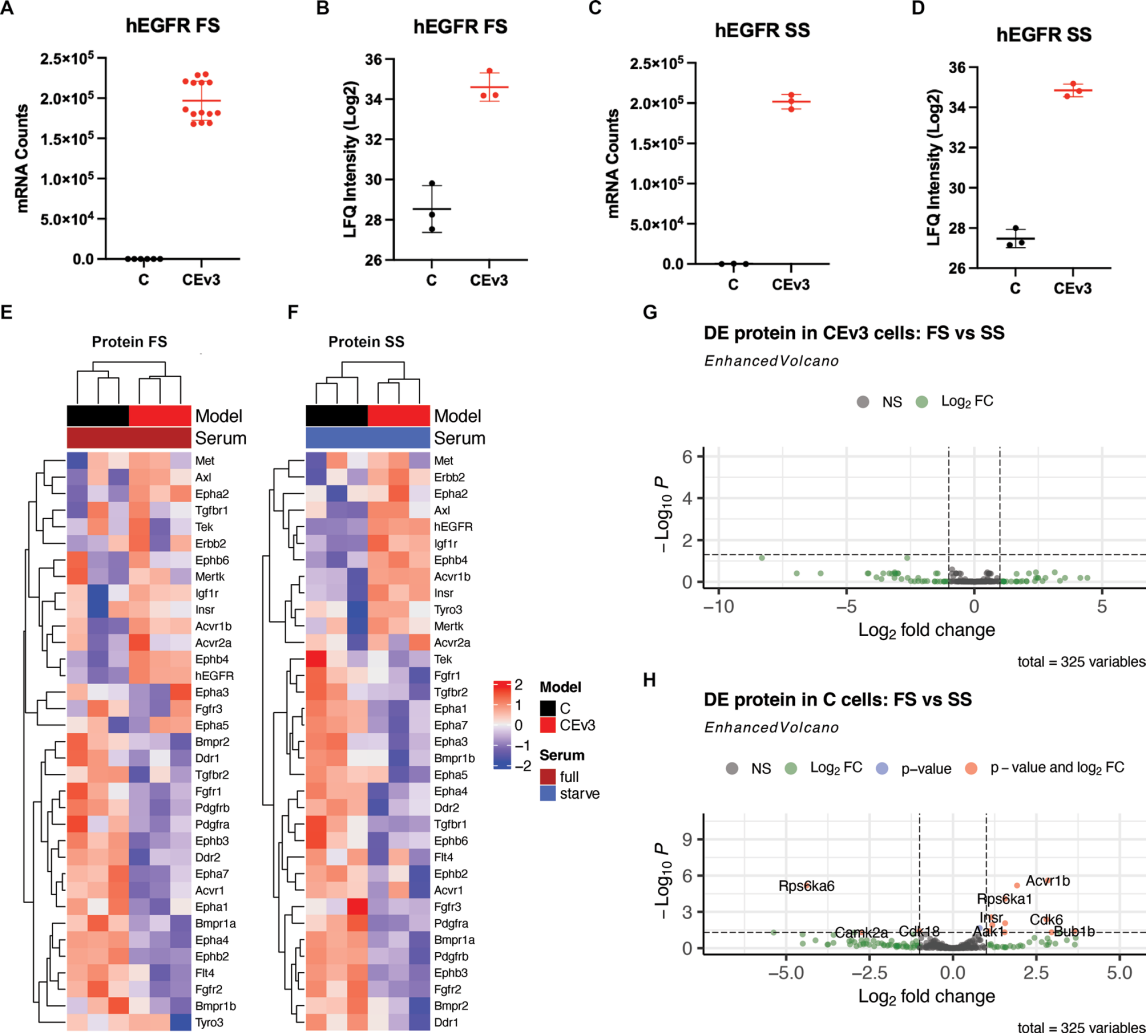
*EGFRvIII (hEGFR) overexpression reprograms receptor kinases*. Cells were cultured in full (FS) or starved serum (SS) conditions. hEGFR was exogenously overexpressed at both the mRNA (**AC**) and protein (**BD**) levels specifically in CEv3 cells. EGFR expression in these cells was independent of culture conditions, whether in full (**AB**) or starved (**CD**) conditions. Unsupervised hierarchical clustering heatmaps of kinase receptors detected in C and CEv3 cells show consistent downregulation of these receptors at the protein level in both full (**E**) and starved (**F**) culture conditions. Volcano plots showing DE kinase proteins between full and starved serum culture conditions for CEv3 (**G**) and C cells (**H**)

CEv3 cells had significantly increased hEGFR mRNA levels (q < 0.05 by DESeq2) (Fig. 1A) and a ~ 32-fold (q < 0.05 by limma) increase in protein levels (Fig. 1B) compared to parental C cells. Culture in starved serum did not affect mRNA (Fig. 1C) or protein (Fig. 1D) expression levels of hEGFR. The relatively low levels of hEGFR detected in murine C cells that lack hEGFR expression were likely an artifact of improper spectral matching between runs. Failure to detect high hEGFR reads by RNA-seq supports this conclusion. The low number of mapped hEGFR reads in C cells is likely due to sequence homology between human and mouse EGFR.

We next used protein and mRNA expression levels of receptor kinases to monitor EGFRvIII proteogenomic programming. There are fifty-eight (58) receptor tyrosine kinases and twelve (12) receptor serine/threonine kinases in humans that can initiate canonical signaling, including MAPK, JAK/STAT, and PI3K [29]. These effector pathways govern fundamental cellular processes and are remarkably plastic [12, 29, 44]. Unsupervised hierarchal clustering with all captured receptor kinases showed that CEv3 cells have a distinct receptor kinase proteome compared to C cells when cultured in full serum (Fig. 1E). Since EGFRvIII is constitutively active and cannot bind extracellular ligand, we also analyzed the receptor kinase proteome upon serum starvation (Fig. 1F). The CEv3 kinome remained unaltered, as no DE kinases were identified between full and serum starved conditions (Fig. 1G). hEGFR expression induced a unique proteomic program with striking changes in receptor kinase protein levels.

In contrast, parental C cells exhibited DE of several receptor kinases between culture conditions (Fig. 1H). Serum provides extrinsic stimuli in the form of growth factors and ligands that bind and activate receptor kinases. These data demonstrate that the plasticity of the kinase proteome in CEv3 cells in response to extracellular stimuli is less dynamic than that of the kinase proteome in C cells, suggesting a dominant role for EGFRvIII in shaping the GBM kinome landscape.

We next evaluated whether hEGFR expression induced similar changes in the receptor kinase transcriptome. Unsupervised hierarchical clustering of receptor kinase genes from RNA-seq shows separation between CEv3 and C cells in both full serum (Fig. S2A) and starved serum culture conditions (Fig. S2B). A direct comparison of the entire transcriptome for each cell line and culture condition revealed few DE genes in CEv3 cells (Fig. S2C), in contrast to the many DE genes in C cells (Fig. S2D). These data align with MIB-MS results at the protein level and suggest that hEGFR overexpression induces reprogramming of the receptor kinase transcriptome landscape, thereby suppressing changes across the transcriptome resulting from serum deprivation. Thus, CEv3 cells provide an excellent platform to model the EGFRvIII GBM kinome.

### The hEGFR-driven kinome is reprogrammed upon acquired TKI resistance

We serially passaged CEv3 cells with increasing concentrations of erlotinib (E4 and E5) or gefitinib (G1, G5, G8, and G12) to generate cells with acquired EGFR TKI resistance (Fig. S1B) [51] We previously demonstrated that these resistant cells showed significantly increased in vitro and in vivo proliferation compared to CEv3 cells when treated with EGFR TKI [51]. We therefore performed MIB/MS and RNA-seq on drug-resistant CEv3 cells to further elucidate kinome dynamics after acquiring TKI resistance.

**Fig. 2:**
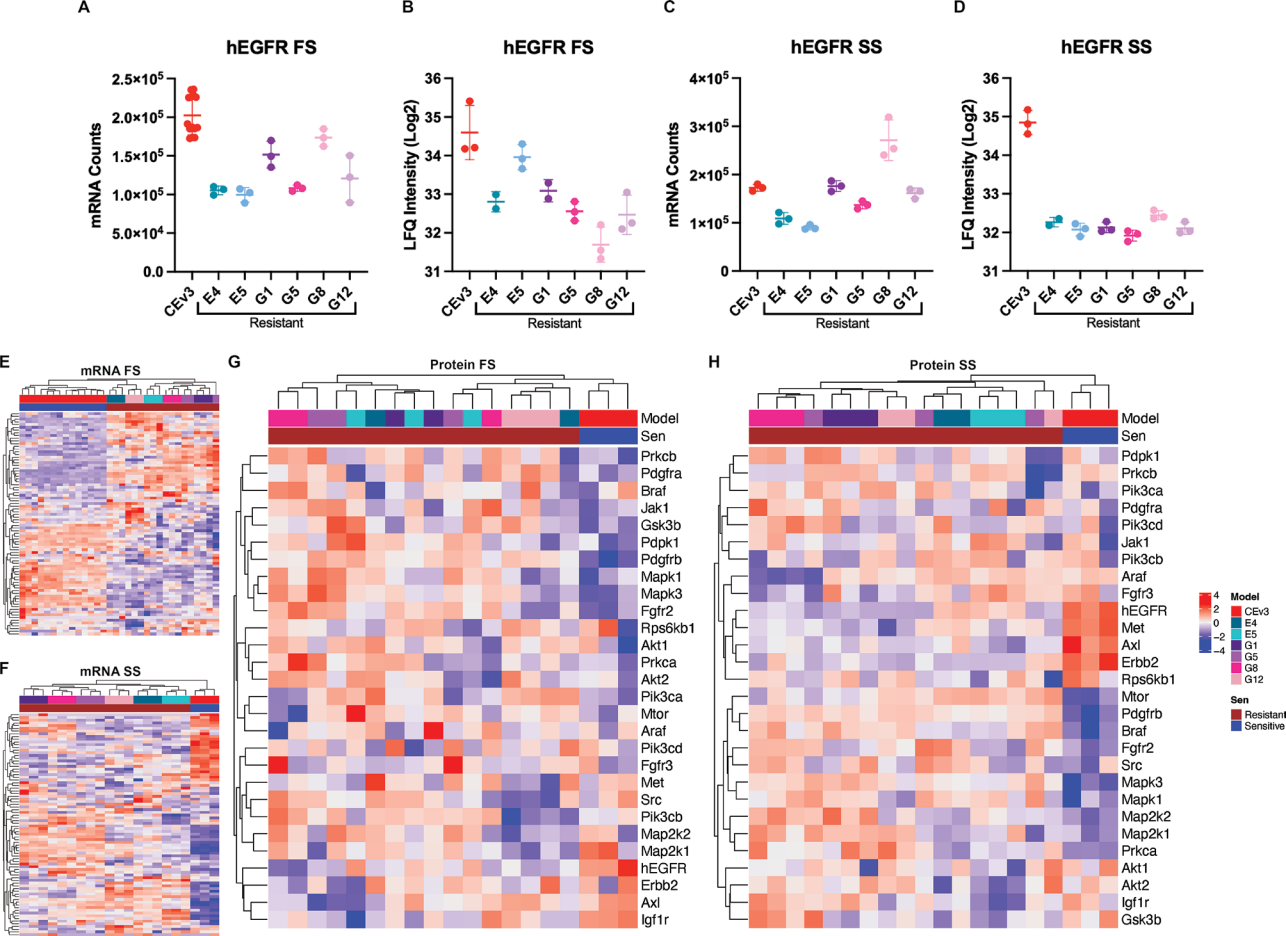
*Chronic inhibition of hEGFR rewires the kinome*. Decreased hEGFR expression is evident in resistant models E4, E5, G1, G5, G8, and G12. hEGFR mRNA (**AC**) and protein (**BD**) is reduced in resistant models in both full (**AB**) and starved (**CD**) serum conditions compared to the parental CEv3 model. Unsupervised hierarchical clustering of genes associated with EGFR TKI resistance (MSigDB WikiPathway [1]) reveals that resistant cells dynamically rewire their kinase transcriptomes (**EF**) and proteomes (**GH**), regardless of whether they are cultured under full (**EG**) or starved (**FH**) conditions

Resistant cells exhibited decreased hEGFR mRNA (Fig. 2A and C) and protein (Fig. 2B and D) levels compared to parental CEv3 cells, regardless of whether they were cultured in full (Fig. 2A and B) or starved serum (Fig. 2C and D). To understand how EGFR TKI resistance resulting from chronic drug exposure rewires the hEGFR kinome, we performed unsupervised hierarchical clustering using genes from the Wikipathway EGFR TKI resistance signature [1]. This published gene signature contains eighty-two (82) genes, including receptor kinases, ligands, and signaling mediators associated with resistance in breast cancer, lung cancer, and GBM cell lines. A heatmap of mRNA levels of this gene signature separated CEv3 cells from their drug-resistant counterparts, whether they were cultured in full (Fig. 2E) or starved serum (Fig. 2F). Similarly, a heatmap of protein levels of kinases from MIB/MS using the same resistance signature separated CEv3 cells from their EGFR TKI-resistant counterparts in both full (Fig. 2G) and starved serum (Fig. 2H) cultures.

Pdgfrβ and Mapk3, established genes involved explicitly in acquired EGFR TKI resistance in GBM, were upregulated in resistant cell lines. Chronic EGFR TKI exposure induced upregulation of Pdgfrβ in all resistant lines at both mRNA (Fig. S3A and C) and protein levels (Fig. S3B and D) regardless of culture condition. EGFR TKI-induced transcriptional derepression of PDGFRβ has been previously observed [2]. Wykosky et al. have shown that adaptive bypass signaling via Mapk3 drives acquired resistance in these models [51]. Similarly, we detected Mapk3 upregulation in all resistant cells at both mRNA (Fig. S3E and G) and protein levels (Fig. S3F and H). Importantly, this upregulation occurs in both culture conditions, suggesting that Mapk3 is intrinsically upregulated in the absence of ligand-induced stimulation. Within the resistant cells, we also observed heterogeneity in expression of components of this gene signature (Fig. 2G and H). For example, Fgfr2 exhibits heterogeneous upregulation in EGFR TKI-resistant CEv3 cells (Fig. S3E-H).

Unsupervised hierarchical clustering heatmaps (Fig. 2E-H) show proteogenomic heterogeneity in the EGFR TKI resistance gene signature among resistant lines. Examination of kinase mRNA and protein levels in this gene signature reveals evidence of hEGFR kinome rewiring (Fig. S3). This rewiring likely contributes to acquired drug resistance. This kinomic heterogeneity persists in both full and starved serum conditions, suggesting that kinome rewiring during the acquisition of drug resistance may be a stochastic process. To investigate this, we sought to identify altered kinase signatures, both unique and shared, associated with each EGFR TKI-resistant cell line when compared to CEv3 cells.

### Kinomic profiling demonstrates cell line-specific EGFR TKI-induced kinome rewiring

**Fig. 3:**
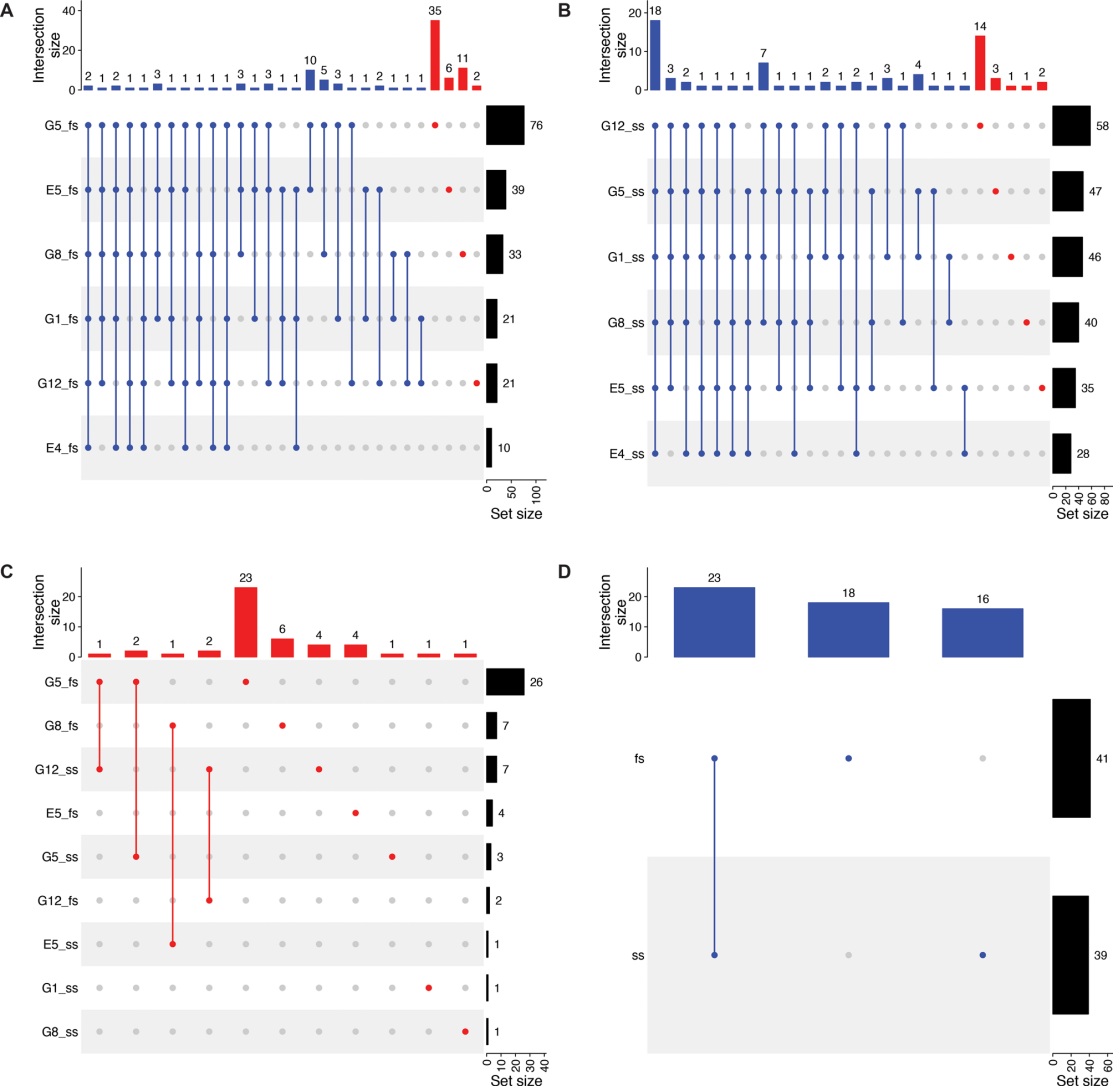
*Unique kinase signatures define clones emerging after chronic EGFR TKI treatment*. Few DE kinases (limma FDR < 0.05) are shared (blue) among resistant clones cultured in either full (**A** and Table ST. 1) or starved (**B** and Table ST. 2) serum conditions. These DE kinases are unique to each clone and culture condition, except for three genes in the G12 signature (**C** and Table ST. 3). Twenty-three (23) DE kinases are shared by at least two resistant clones in both culture conditions (**D** and Table ST. 4)

To identify signatures associated with kinome rewiring induced during acquired resistance, we first identified DE kinases from proteomics data. We compared each resistant cell line with parental CEv3 cells, cultured in either full (Fig. S4) or starved serum (Fig. S5) conditions, to identify DE kinases (FDR < 0.05). Between ten (10) and seventy-six (76) such kinases were identified in each resistant cell line (Fig. 3A) cultured in full serum. Forty-nine (49) kinases were DE in at least one other cell line, and fitty-four (54) were uniquely DE in a single line (Fig. 3A and Table ST. 1). Twenty-eight (28) to fifty-eight (58) DE kinases were identified in each resistant cell line (Fig. 3B) when cultured in starved serum. Fifty-four (54) kinases were DE in at least one other cell line, and twenty-one (21) were uniquely DE in a single line (Fig. 3B and Table ST. 2). After removing kinases that were labeled “unique” (red) in one culture condition and “shared” (blue) in another, comparisons of DE kinases between full serum and starved serum culture conditions revealed that most were unique to both the resistant cell line and culture conditions (Fig. 3C and Table ST. 4). Thus, we aggregated the “unique” DE kinases in full (fs) and starved (ss) conditions to define baseline (BL) kinase signatures of E5, G1, G5, G8, and G12 cells, as E4 lacked unique DE kinases. To further investigate “shared” DE kinases, we limited the overlapping (OL) BL signature only to kinases up- or downregulated in either culture condition to eliminate DE kinases associated with extrinsic cellular factors (Fig. 3D). We identified twenty-three (23) kinases that met these filtering criteria, constituting an OL BL kinase gene signature (Table ST. 5). These BL signatures, both OL and unique, enable us to refine our kinomic analysis further. We used these signatures to determine how each resistant cell line reprograms their kinome after acquiring drug resistance.

**Fig. 4:**
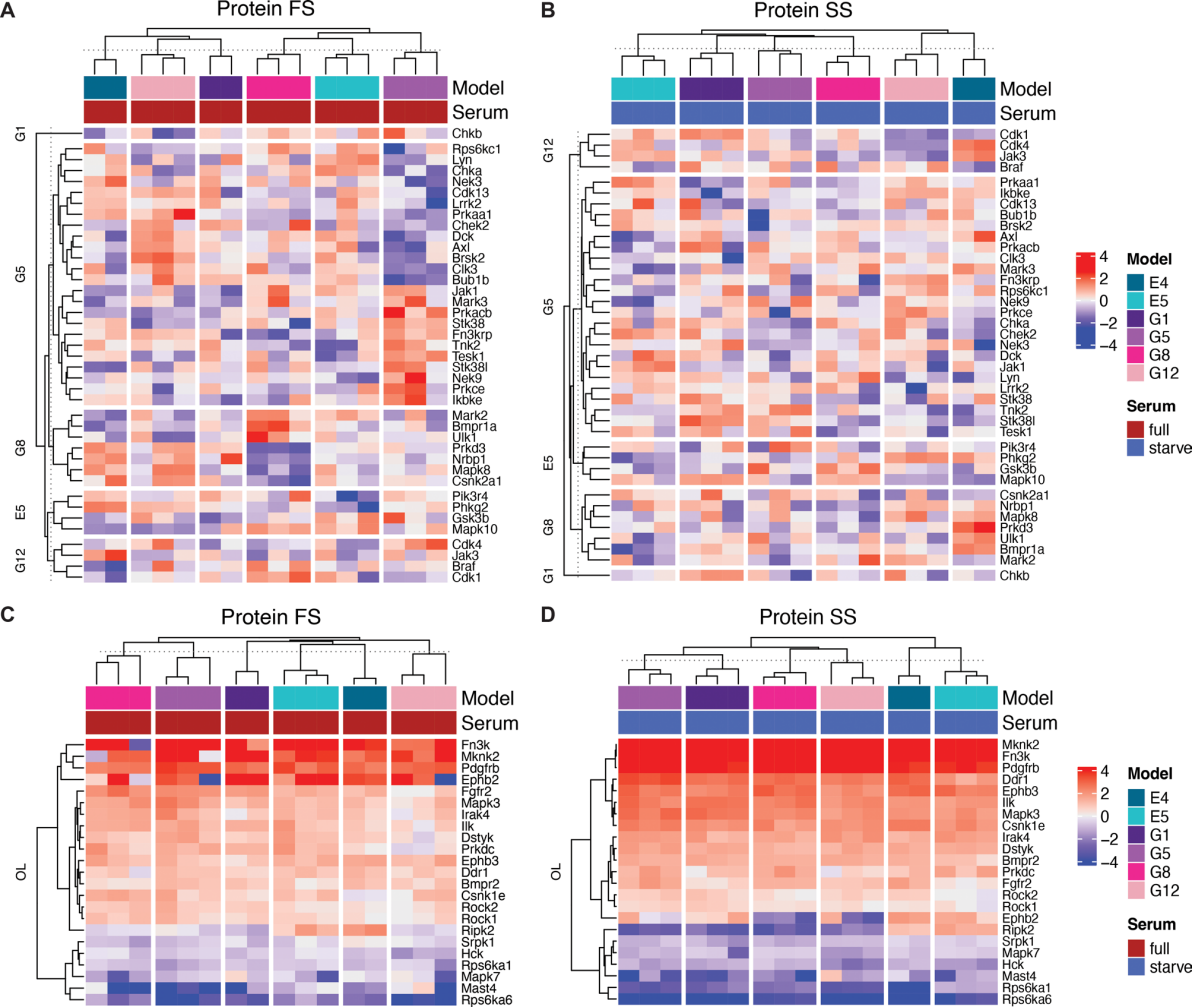
Supervised hierarchal clustering discriminate unique and shared DE kinases after chronic EGFR TKI treatment. Supervised hierarchical clustering was performed with baseline kinase signatures (Table ST. 5) from normalized protein levels of genes in each unique baseline signature (**AB**) and the overlapping (**OL**) baseline signature (**CD**) in full (**AC**) and starved serum conditions (**BD**). Normalized protein for each cell line was generated by subtracting the mean gene protein level for CEv3 replicates from each gene for every replicate. Normalized values were limited to the − 4,4 range and visualized without center-scaling (**C**, **D**)

Supervised hierarchical clustering with these kinase signatures (Table ST. 5), collectively referred to as baseline (BL) signatures, shows that the unique DE kinase signatures can discriminate resistant cell lines. Heatmaps generated from protein levels normalized to CEv3 cells show unique enrichment of each signature with respect to the cell line in full serum (Fig. 4A) and starved serum (Fig. 4B) conditions. Generally, kinases in the OL BL signature are either upregulated or downregulated in all resistant cell lines compared to CEv3. Heatmaps generated from clipped protein levels normalized to CEv3 show a homogenous supervised clustering pattern in all resistant cell lines in full serum (Fig. 4C) and starved serum (Fig. 4D) conditions. These DE signature kinases identified from proteomics have matched changes in mRNA levels as determined by transcriptomics in both full (Fig. S5A) and starved (Fig. S5B) culture conditions. Although there were few signature kinases that were identified as DE at the mRNA level, there was a moderate to strong (*R* = 0.5–0.7) Pearson correlation between changes in the protein level with changes in the mRNA level for each resistant cell line in both culture conditions. These data suggest that transcriptomic characterization alone of kinome rewiring may underrepresent the scale of the functional kinase proteome which may be regulated by post-translational modification.

This extensive characterization of kinome dynamics in models of acquired resistance to EGFR TKIs provides potential candidates for combination therapies. However, only a subset of kinase reprogramming was consistent across multiple resistant cell lines derived from the same parental cell line. The heterogeneity of kinome reprogramming represents a significant obstacle in the design of combination treatment strategies after resistance has emerged. Kinome reprogramming has been observed in other cancers in as little as 24 h of drug treatment [18, 26, 34, 36]. Thus, we sought to more fully characterize the response of the kinome to EGFR TKIs in the acute phase. By studying the acute response to EGFR TKI, we aim to identify candidates for upfront combination therapy that overcome the complications introduced by the heterogeneity of kinome dynamics in acquired resistance.

### EGFR TKI induce distinct acute kinomic rewiring

We aim to design therapeutic strategies informed by the lessons learned from the failures of early GBM EGFR clinical trials, as we highlighted previously [24]. EGFR TKI has failed to prolong median overall survival as both a single agent and as an adjuvant therapy in GBM [24]. We speculate that upfront co-inhibition of EGFR and a TKI-upregulated second kinase will be therapeutically synergistic. We prioritize kinases that are acutely active in kinome rewiring (a yet to be defined EGFRi signature) and altered in acquired resistance (BL kinase signatures defined in Table ST. 5). We speculated that the acute kinome response to EGFR TKI would be more uniform than the heterogenous responses observed with chronic acquired resistance and would yield potential targets for combination therapy. We further speculated that upfront co-inhibition of EGFR and dynamically regulated kinases would be therapeutically beneficial by preventing acquired resistance to single-agent EGFR TKI. Comparisons between BL kinase signatures (Table ST. 5) and this EGFR inhibitor acute signature (EGFRi) may provide further insight into the heterogeneity of acquired resistance.

**Fig. 5:**
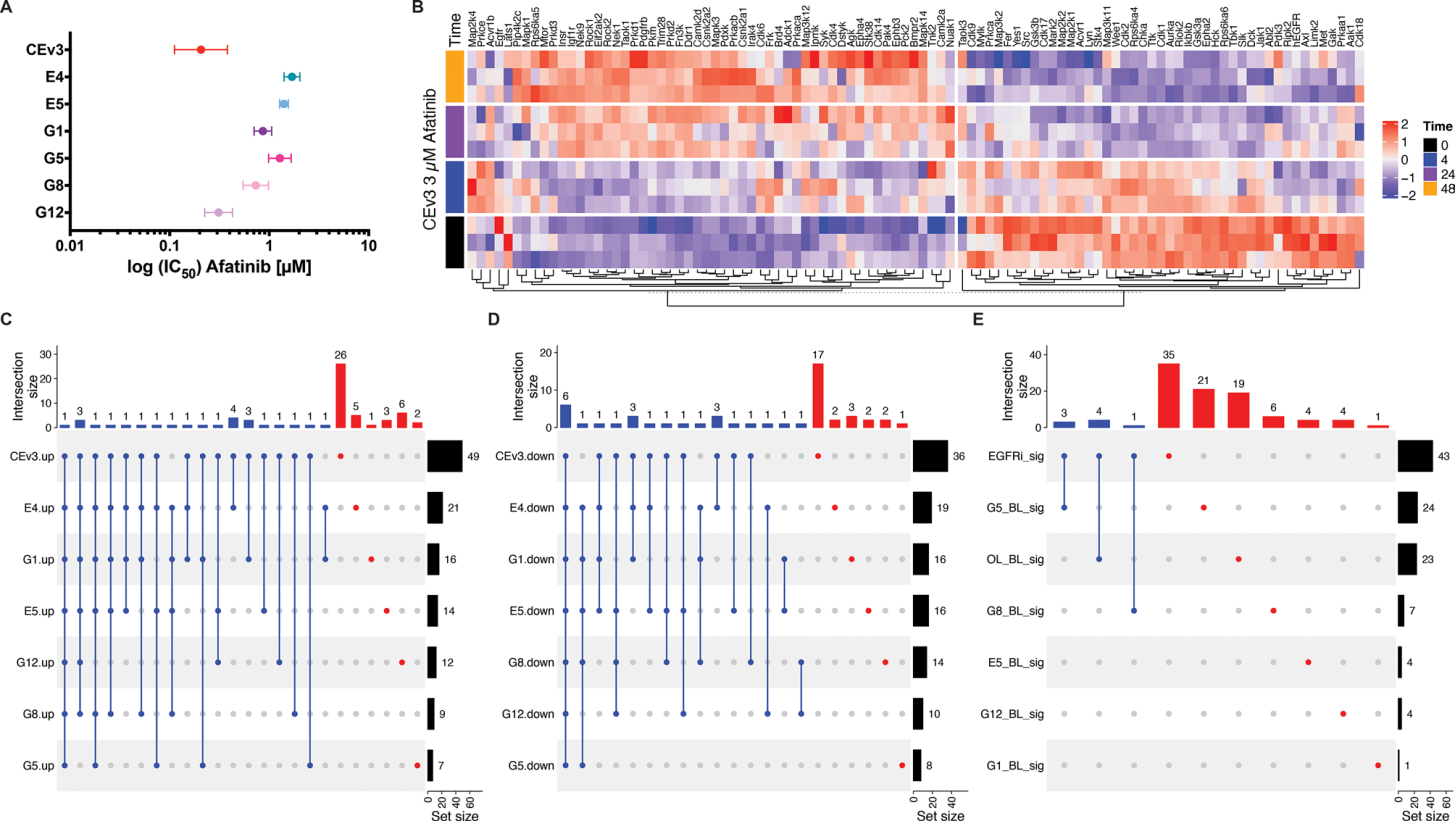
Dynamic kinase rewiring occurs acutely after EGFR TKI treatment. Resistant clones have increased IC_50_ to the second-generation EGFR TKI afatinib compared to parental CEv3 cells (*n* = 2 biological replicates with 6 technical replicates) after 72 h of drug treatment (**A**). K-means clustering (**B**) identifies 49 “up” (C and ST. 6) and 36 “down” (D and Table ST. 7) DE kinase proteins (limma FDR < 0.05) in CEv3 cells. A small subset of these was also identified in resistant clones that do not express hEGFR. DE kinases unique to CEv3 constituted the EGFRi signature (EGFRi_sig) and represent those that dynamically rewire within 48 h of drug inhibition. The EGFRi_sig is distinct from baseline kinase signatures (labeled “BL_sig”) previously identified from drug-resistant clones (5 F and Table ST. 8)

To identify the acute EGFRi kinome signature, we performed multiplexed Tandem Mass Tag (TMT) MIB-MS profiling before and after 4, 24, and 48 h of treatment with the second-generation EGFR inhibitor afatinib in parental CEv3 and resistant cell lines. Erlotinib- or gefitinib-resistant cells were cross-resistant to afatinib (Fig. 5A), as these cells had higher afatinib IC_50_ at 72 h compared to parental cells. To generate the acute EGFRi signature (ST.8) bioinformatically, we first identified DE kinases after challenging both our resistant (E4, E5, G1, G5, G8, and G12) (Fig. S8A-F) and sensitive (CEv3) (Fig. 5B) cells with afatinib. DE kinases identified with limma (FDR < 0.05) for each cell line were classified as “up” or “down” across the 48 h of drug treatment using K-means clustering with 2 clusters (Fig. 5B and S8) after removing kinases with ambiguous directionality. Dynamic kinase signatures were generated for each cell line (Fig. 5B and S8). As expected, CEv3 cells exhibited the most active kinome rewiring, as defined by DE kinases, within 48 h of afatinib treatment (Fig. 5C and D).

Our resistant models have disrupted kinomic programming due to chronic EGFR TKI exposure and express genetic hallmarks of acquired resistance at both the mRNA and protein levels (Fig. 2E-H and S3). Therefore, we reasoned that DE kinases identified in resistant models that were rechallenged with afatinib are less likely to be associated with acute EGFR TKI-induced kinome rewiring. Twenty-six (26) kinases showed increased expression uniquely in CEv3 cells (Fig. 5BC and Table ST. 6), while seventeen (17) kinases were decreased (Fig. 5D and Table ST. 7) after afatinib treatment. Collectively, these forty-three (43) kinases composed the EGFRi signature (Table ST. 8), which had minimal overlap with the previously identified BL resistant cell signatures (Fig. 5E and Table ST. 9). Only four kinases from the EGFRi signature were shared with the OL_BL signature and eight kinases were shared between the acute EGFRi and acquired resistance BL signatures (Fig. 5E and Table ST. 9). These data suggest that acute kinome rewiring is distinct from kinome rewiring after acquired resistance develops from chronic drug exposure.

The lack of similarities in the kinomic signatures established between acute and chronic drug responses demonstrates the plasticity of the GBM kinome in response to EGFR TKI. The data suggest that precision oncology approaches targeting molecular vulnerabilities identified in resistant cell lines may not address the acute kinome rewiring observed in the initial drug response in GBM.

### EGFRi kinase signature shows an unexpected increase in Cdk6 protein levels

**Fig. 6:**
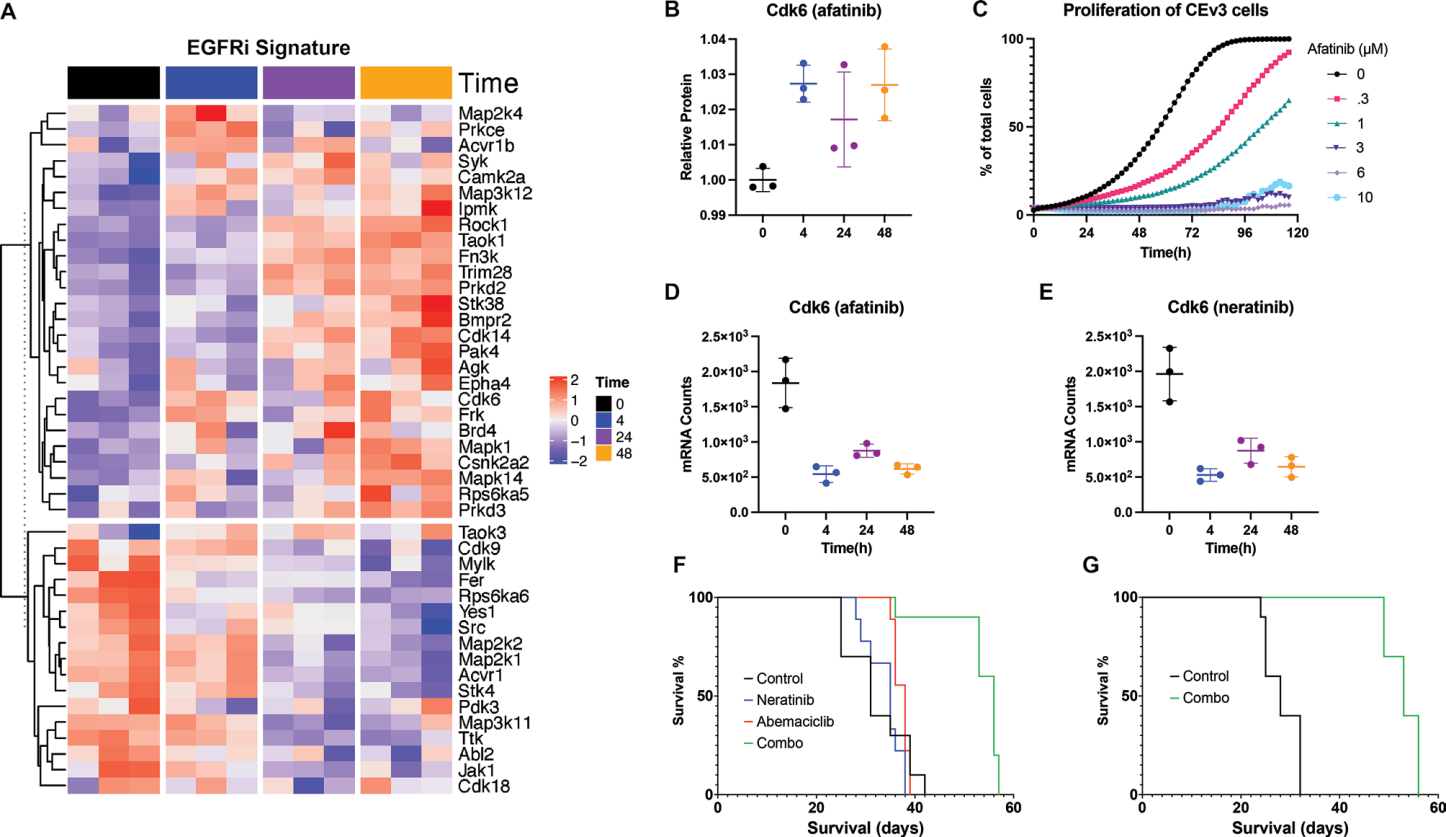
Upfront targeting of EGFRvIII and Cdk6 provides a combinatorial synthetic lethality. The EGFRi signature define DE kinases associated with acute EGFR TKI treatment (**A**). Afatinib treatment induces a significant (limma FDR < 0.05) increase of Cdk6 protein levels within 48 h (**B**). Log2-transformed TMT protein intensities were normalized to the no treatment controls at 0 h. Afatinib induced a dose-dependent decrease in CEv3 proliferation, as observed using live cell imaging (**C**). Afatinib (**D**) and neratinib (**E**) decreased Cdk6 mRNA levels over a 48-hour period. Dual therapy with neratinib and abemaciclib extends median overall survival of mice (*n* = 10 mice per treatment arm) bearing orthotopic CEv3 allografts from 31 days (untreated controls) to 56 days, while neither single agent is effective. Treatment with the respective therapy commenced 1-week post-injection for 6 weeks (**F**). A second experiment showed a similar survival benefit of combination treatment continued past 6 weeks (**G**)

Kinomic adaptations to targeted therapy are not unique to GBM. Several other cancers exhibit acute rewiring that renders single agents ineffective [18, 26, 34, 36]. Whereas single-agent kinase inhibitors have failed in these cancers, targeting kinome remodeling events upfront has proven effective [18, 26]. To identify upfront combinatorial targets in EGFRvIII-driven GBM, we focused on the 26 kinases in the EGFRi signature that increased their expression after TKI treatment (Fig. 6A and Table ST. 8). Among these twenty-six (26) kinases, three interaction networks were identified upon functional protein association network analysis using STRING-DB (Table ST. 8) [41, 46]. The largest network consisted of genes associated with mitogenic signaling pathways. Two kinases from the protein kinase D family formed another network. The last contained four (4) genes, including the cell-cycle-associated kinase Cdk6. Cdk6 protein levels increased significantly in CEv3 cells upon afatinib treatment (Fig. 6B). Upregulation of a kinase that controls cell cycle progression was unexpected as afatinib inhibited proliferation of CEv3 cells (Fig. 6C). To further investigate the unexpected increase in Cdk6 protein with decreased cell growth, we transcriptionally profiled CEv3 cells after treatment with afatinib or neratinib. Neratinib is an irreversible TKI of HER2/ERBB2 and other EGFR family receptors that has been used in clinical trials for EGFR-positive GBM [3]. The transcriptomes of CEv3 cells were sequenced before and after 4, 24, or 48 h of drug treatment. Interestingly and in contrast to protein levels of Cdk6, mRNA levels of Cdk6 decreased significantly after drug treatment (Fig. 6D and E) suggesting post-translational stabilization or increased activation of Cdk6 and enhanced MIB binding in response to EGFR TKI.

To gain further insight into the altered biological processes in EGFR TKI-treated CEv3 cells, we directionally labeled DE genes identified using k-means clustering (Fig. S10A, B). To avoid off-target effects resulting from differing kinase specificities between afatinib and neratinib, only DE genes with the same direction of change in response to either drug were considered. This included upregulation of 980 and downregulation of 844 genes (Fig. S10C). Functional enrichment analysis of this DE gene signature using gProfiler2 revealed upregulated stimulus response, cell motility, and cell surface receptor pathways (Fig. S10D). Cell cycle progression and mitosis pathways were among the top downregulated pathways (Fig. S10E). The transcriptomic and drug dose-response data provide orthogonal validation that EGFR TKI decreased the proliferation of CEv3 cells, despite the increase in Cdk6 protein identified by proteomics. We therefore sought to therapeutically exploit this drug-induced increase in functional Cdk6 protein through upfront combinatorial therapy with EGFR TKI.

### Dual inhibition of hEGFR and Cdk6 prolongs survival in vivo

To select drugs for an in vivo survival study, we prioritized FDA-approved drugs for both EGFR and CDK6. Neratinib was selected to inhibit hEGFR since it was in clinical trials for patients with EGFR-positive GBM [3]. There are no Cdk6-specific inhibitors in clinical trials or approved by the FDA. Thus, we used the Cdk4/6 inhibitor abemaciclib, which is approved for use in breast cancer patients. We generated orthotopic CEv3 allografts and randomized ten (10) mice into each of four treatment arms to test if combination neratinib and abemaciclib provided an enhanced survival benefit over single-agents. Cohorts of allografted mice were treated with neratinib, abemaciclib, neratinib and abemaciclib, or vehicle control. No significant difference in median survival (Mantel-Cox) was observed in neratinib (35 days) or abemaciclib (38 days) groups compared to control (31 days) (Fig. 6F). However, the combination of neratinib and abemaciclib almost doubled median survival (56 days vs. 31 days, Mantel-Cox *p* < 0.0001). The survival benefit of combination therapy was validated in a second cohort of orthotopic allografts (Fig. 6G) where median survival almost doubled (53 days vs. 28 days, Mantel-Cox *p* < 0.0001). These data demonstrate that the upfront combination of neratinib and abemaciclib provide a synergistic survival benefit.

## Discussion

Acquired resistance is a significant obstacle to precision oncology approaches. Several acquired resistance mechanisms specific to EGFR-driven GBM have been identified [7, 35, 53]. Here, we characterized TKI-induced kinome rewiring in a preclinical model of EGFRvIII-driven GBM both acutely and after acquired resistance had developed [2, 19, 24]. We identified a shared overlapping (OL BL) kinase signature as well as unique cell line specific signatures from DE kinase proteins in resistant lines (Table ST. 5). Protein and mRNA levels of kinases in the BL signature significantly correlated, suggesting that kinome rewiring is regulated at multiple levels from transcription to post-translational modifications (Table ST. 6 and ST. 7). While we did not explore epigenetics here, the transcriptional changes observed may be governed by concordant epigenetic changes through promoter/enhancer remodeling and/or histone acetylation/methylation, as previously described [25]. Indeed, we identified the epigenetic transcriptional regulator Brd4 as an upregulated member of the EGFRi signature (Fig. 6A and S9 and Table ST. 8). Future studies will be needed to investigate the epigenetic contributions to kinome rewiring after TKI treatment in EGFR-driven GBM.

The rewired kinome after acquired resistance was heterogeneous. This presents obstacles for sequential kinase inhibition strategies. Our group has previously shown that inhibition of Mapk3 restores sensitivity to EGFR TKI in resistant models and our data here classifies Mapk3 as a shared upregulated kinase in acquired resistance cells (Fig. S3E-H) but not in the acute EGFRi signature (Fig. 6A). Therefore, it is unclear if long-term exposure to dual therapy would produce a durable response or if the plasticity of cellular signaling pathways would further rewire to compensate. Instead, we sought to replicate the success that other neoplasms have had with upfront combinatorial therapy. By characterizing the acute kinome rewiring induced by EGFR TKI, we aimed to uncover a kinase signature shared between acute rewiring and acquired resistance that can be therapeutically exploited in the upfront combinatorial therapy setting.

We defined an EGFRi signature, a set of kinases with significantly altered functional protein expression level after inhibition of hEGFR within 48 h. Interestingly, few kinases were shared between the EGFRi and baseline kinase signatures (Fig. 5E). These data indicate that kinome rewiring during the acute phase differs from that in acquired resistance. Considering the temporal plasticity we observed in drug-induced EGFRvIII kinome rewiring, we abandoned attempts to inhibit shared targets in EGFRi and baseline kinase signatures. Instead, we focused on identifying a target from the EGFRi signature that would provide a mechanistically orthogonal synthetic lethality with EGFR TKI in the upfront combinatorial therapy setting. Further molecular interrogation of the 26 EGFRi signature kinases with increased expression after drug treatment revealed an unexpected increase in Cdk6 protein, despite a decrease in proliferation (Fig. 6BC). We hypothesized that we could exploit this increase in Cdk6 as a synthetically lethal target with hEGFR. Our orthotopic allograft survival studies confirmed a synergistic increase in survival when hEGFR and Cdk6 are targeted simultaneously (Fig. 6FG). These results show that the rational design of upfront dual-targeted combination therapy based on kinome reprogramming can be an effective therapeutic strategy.

Our molecular studies show upregulation of Cdk6 protein but not mRNA after EGFR inhibition, suggesting transcription-independent regulatory mechanisms. Cdk6 is activated post-translationally through phosphorylation-dependent binding to Cyclin-D (Ccnd1). Our MIB-MS proteomics assay captures functional levels of Cdk6 and serves as a proxy for activity. However, future studies should investigate Cdk6 activation through post-translational modifications or protein stability to elucidate the regulatory mechanisms that contribute to the discordance between mRNA and protein levels observed in this study. Alternatively, the increase of Cdk6 protein after EGFR inhibition may provide negative feedback to decrease Cdk6 mRNA levels. Future studies focused on Cdk6 mRNA transcription and stability are needed to understand post-transcriptional regulation of Cdk6 in the context of EGFR inhibition.

While our study identified a synthetic lethality target in a preclinical model of GBM, it is not without limitations. Genetically engineered mouse astrocyte models enable precise characterization of genotype-phenotype relationships, but they fail to accurately model other aspects of EGFR-driven GBM. EGFRvIII mutations frequently co-occurs with amplification of wild-type EGFR on extrachromosomal DNA (ecDNA) [24]. We have not addressed other genomic mechanisms of EGFR TKI resistance, such as ecDNA. Moreover, the genetically engineered astrocyte model (C cells) is from a mixed genetic background and constitutive overexpression of the human EGFRvIII (hEGFR) oncogene is exogenously induced. While we have shown that dual upfront inhibition of EGFRvIII and Cdk6 significantly prolongs survival, these limitations must be addressed before testing this therapeutic approach clinically. Next-generation genetically engineered models derived from human induced pluripotent stem cells (iGBM) and patient-derived tumor models overcome many of these limitations [20, 32]. Next-generation iGBM models generated by our group enable the in vitro and in vivo therapeutic characterization of GBM in both genetically homogeneous and heterogeneous EGFR-driven backgrounds. Patient-derived xenografts (PDX) faithfully recapitulate the complex genetics observed in patient tumors and retain the transcriptomic profiles of the original tumors [14, 49]. To accurately identify clinically actionable synthetic lethality targets for combination therapy in EGFR-driven GBM, future validation studies need to characterize the kinomes of genetically matched PDX models treated with novel, brain-penetrant TKI.

Despite these limitations, this study successfully identified synthetically lethal vulnerabilities that are susceptible to combinatorial therapies in a preclinical model of GBM. We developed and utilized an extensive kinome characterization pipeline to identify dyanmic kinase signatures of EGFR TKI response. Thus, we leveraged in vitro and in vivo experimentation in conjunction with bioinformatics analysis to identify and exploit a druggable synthetic lethality target with preclinical efficacy. This study has advanced our understanding of EGFR-driven GBM and provides a framework for identifying candidate targets for upfront combinatorial treatments.

## Conclusion

Characterizing kinome rewiring both acutely and after drug resistance develops enables the identification of targets and the development of logical therapeutic strategies. Future studies applying this kinome characterization pipeline to more clinically translatable models of EGFR mutant-driven GBM may identify clinically actionable targets for combination therapies. Identifying and proactively inhibiting a synthetically lethal target in the upfront disease setting is a viable strategy to prolong survival. It must be explored as a therapeutic option in GBM patients. We recommend designing neoadjuvant/adjuvant window of opportunity trials for GBM patients who are positive for mutant EGFR, using next-generation therapeutics targeting EGFR, such as JCN037, an irreversible TKI specifically designed for CNS neoplasms [13, 47]. These trials will permit proper evaluation of EGFR TKI efficacy in GBM and identification of synthetically lethal targets before stochastic adaptive responses develop.

## Electronic supplementary material

Below is the link to the electronic supplementary material.

Supplementary Material 1: Table ST.1 Genes in upset plot (Fig. 3A) of FS culture of acquired resistant cell lines. Table ST.2 Genes in upset plot (Fig.  3B) of SS culture of acquired resistant cell lines. Table ST.3 Genes in upset plot (Fig. 3C) of unique kinases identified in upset plots from Fig. 3A, B. Table ST.4 Genes in upset plot (Fig. 3D) of shared kinases identified in upset plots from Fig. 3AB. Table ST.5 BL kinase signatures identified in cells with acquired resistance. Table ST.6 Genes in upset plot (Fig. 5C) of upregulated kinases after afatinib treatment. Table ST.7. Genes in upset plot (Fig. 5D) of downregulated kinases after afatinib treatment. Table ST.8 EGFRi signature from DE kinases identified in acutely treated CEv3 and acquired resistance cells. Table ST. 9 Genes in upset plot (Fig.  5E) of BL kinase and EGFRi signatures.

Supplementary Material 2: Fig. S1 Model generation. Cdkn2a−/− mouse astrocytes were cultured and transduced with retrovirus encoding human EGFRvIII as described (A) [4, 40]. Resistant models (B) were generated in vitro via dose escalation of EGFR TKI (gefitinib or erlotinib) as described [51]. Figure created with BioRender

Supplementary Material 3: Fig. S2 EGFRvIII (hEGFR) overexpression reprograms the receptor kinase transcriptome. Unsupervised hierarchical clustering heatmaps (AB) and volcano plots (CD) of DE receptor kinases at the mRNA level in both full (A) and starved (B) culture conditions show consistent downregulation in CEv3 (C) and C (D) cells. Full and starved serum cultures are denoted FS and SS, respectively

Supplementary Material 4: Fig. S3 Acquired resistance alters specific kinases. mRNA (AC, EG, IK) and protein (BD, FH, JL) levels of Pdgfr (A-D), Mapk3 (E-H), and Fgfr2 (I-L) in resistant models compared to the parental CEv3 model

Supplementary Material 5: Fig. S4 DE kinases in acquired resistant cell lines cultured in full serum. Volcano plots of DE kinases (limma FDR < 0.05) are shown for the following resistant lines compared to parental CEv3 cells: E4 (A), E5 (B), G1 (C), G5 (D), G8 (E), and G12 (F). Volcano plots show genes upregulated in acquired resistance on the right and genes upregulated in sensitive CEv3 cells on the left

Supplementary Material 6: Fig. S5 DE kinases in acquired resistant cell lines cultured in starved serum. Volcano plots of DE kinases (limma FDR < 0.05) are shown for the following resistant lines compared to parental CEv3 cells: E4 (A), E5 (B), G1 (C), G5 (D), G8 (E), and G12 (F). Volcano plots show genes upregulated in acquired resistance on the right and genes upregulated in sensitive CEv3 cells on the left

Supplementary Material 7: Fig. S6 Kinase signature protein and mRNA levels correlate in drug-resistant cells cultured in full serum. Scatter plots of genes in baseline kinase signatures (Table ST. 5) show significant Pearson correlations between protein and mRNA Log2FC values in the following resistant lines compared to parental CEv3 cells: E4 (A), E5 (B), G1 (C), G5 (D), G8 (E), and G12 (F)

Supplementary Material 8: Fig. S7 Kinase signature protein and mRNA levels correlate in drug-resistant cells cultured in starved serum. Scatter plots of genes in baseline kinase signatures (Table ST. 5) show significant Pearson correlations between protein and mRNA Log2FC values in the following resistant lines compared to parental CEv3 cells: E4 (A), E5 (B), G1 (C), G5 (D), G8 (E), and G12 (F)

Supplementary Material 9: Fig. S8 Afatinib induces acute kinome rewiring in resistant clones. K-means clustering identifies upregulated “up” or downregulated “down” DE kinase proteins after drug treatment. Heatmaps of DE kinases after drug treatment are shown for the following resistant lines compared to parental CEv3 cells: E4 (A), E5 (B), G1 (C), G5 (D), G8 (E), and G12 (F)

Supplementary Material 10: Fig. S9 Interaction of upregulated DE Kinases associated with EGFR inhibition. Functional protein association network analysis using STRIND-DB (https://string-db.org/) shows interactions among upregulated kinases in the EGFRi signature

Supplementary Material 11: Fig. S10 EGFR TKI treatment down-regulates cell cycle progression. Using RNA-seq, we identified DE genes after 4, 24, and 48 hours of afatinib (A) or neratinib (B) treatment. Genes were categorized as upregulated “up” or downregulated “down” by K-means clustering. Of these categorized genes, 980 and 844 are significantly (DESeq2 FDR < 0.05 and LFC > 1) increased and decreased after treatment with either drug (C). Functional enrichment analysis of the shared categorized genes with gProfiler2 shows significant upregulation (D) and downregulation (E) of the indicated top 10 pathways, respectively

## Data Availability

Data is available publicly at the time of publication. Raw sequencing data have been deposited at the Gene Expression Omnibus (GEO) (GSE296151). Public GitHub repositories host pre-processed files (https://github.com/benlin-UAB/2025_Lin_ComboSyntheticLethality_Manuscript) and code used to generate figures (https://benlin-uab.github.io/2025_Lin_ComboSyntheticLethality_Manuscript/) [23].

## Abbreviations

GBM: Glioblastoma
TCGA: The Cancer Genome Atlas
TKI: Tyrosine kinase inhibitor
NSCLC: Non–small cell lung cancer
RTK: Receptor tyrosine kinase
FDA: Federal Drug Administration
DMEM: Dulbecco’s Modified Eagle Medium
FBS: Fetal bovine serum
HPC: High–performance cluster
MIB-MS: Multiplexed inhibitor beads–mass spectrometry
TMT: Tandem mass tags
LFQ: Label–free quantification
IACUC: Institutional Animal Care and Use Committee
hEGFR: Human EGFRvIII
DE: Differentially expressed
FS: Full serum
SS: Starved serum
OL: Overlapping
BL: Baseline
