# Supplementary figures and images for: Identifying and exploiting combinatorial synthetic lethality by characterizing adaptive kinome rewiring of EGFRvIII-driven glioblastoma
Source: Acta Neuropathol Commun. 2025 Jun 28;13:143. doi: 10.1186/s40478-025-02068-y (PMC12205505; doi:10.1186/s40478-025-02068-y)

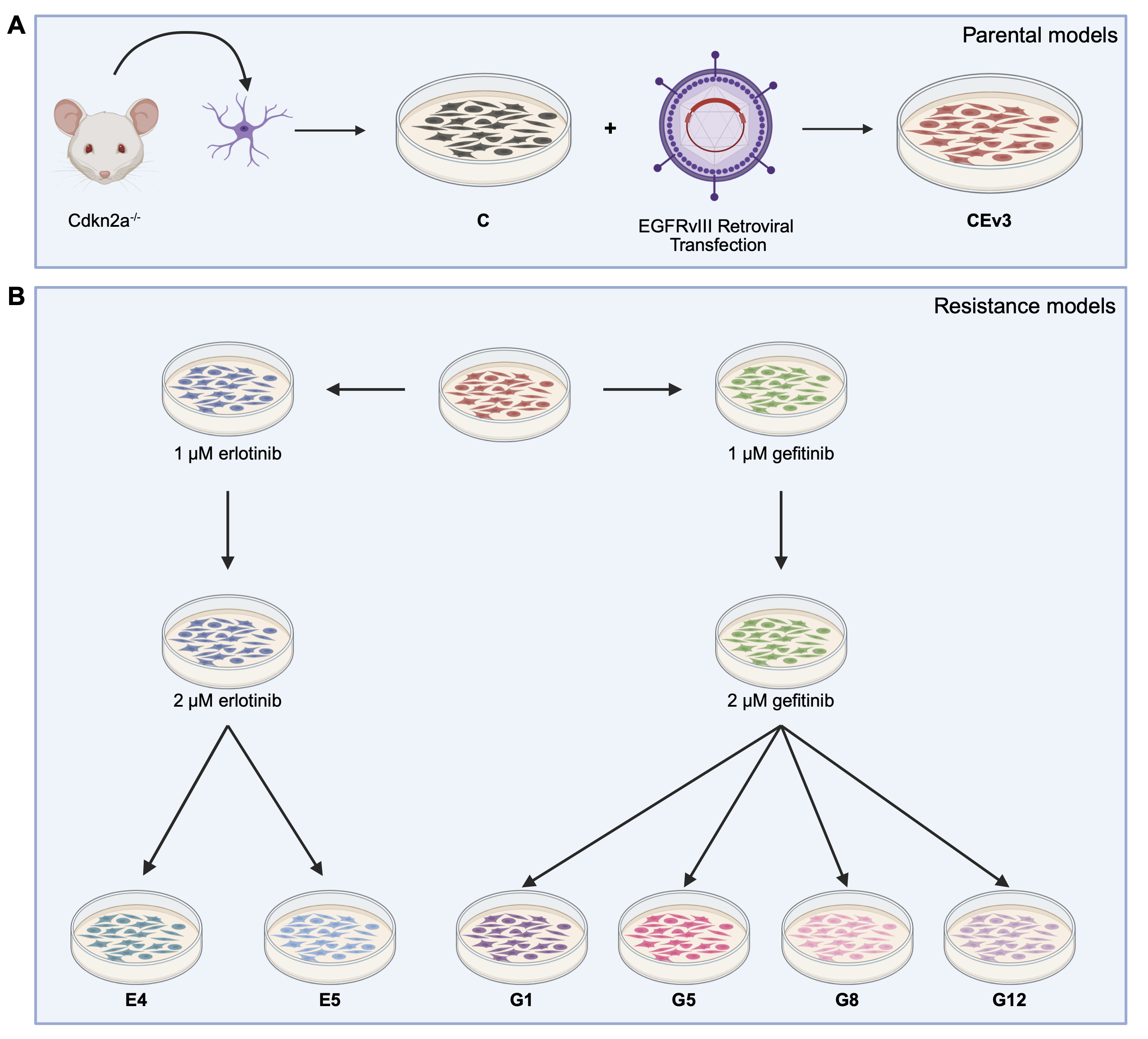

Supplement: Supplementary file 2 — Supplementary Material 2: Fig. S1 Model generation. Cdkn2a−/− mouse astrocytes were cultured and transduced with retrovirus encoding human EGFRvIII as described (A) [4, 40]. Resistant models (B) were generated in vitro via dose escalation of EGFR TKI (gefitinib or erlotinib) as described [51]. Figure created with BioRender [file 40478_2025_2068_MOESM2_ESM.tif]

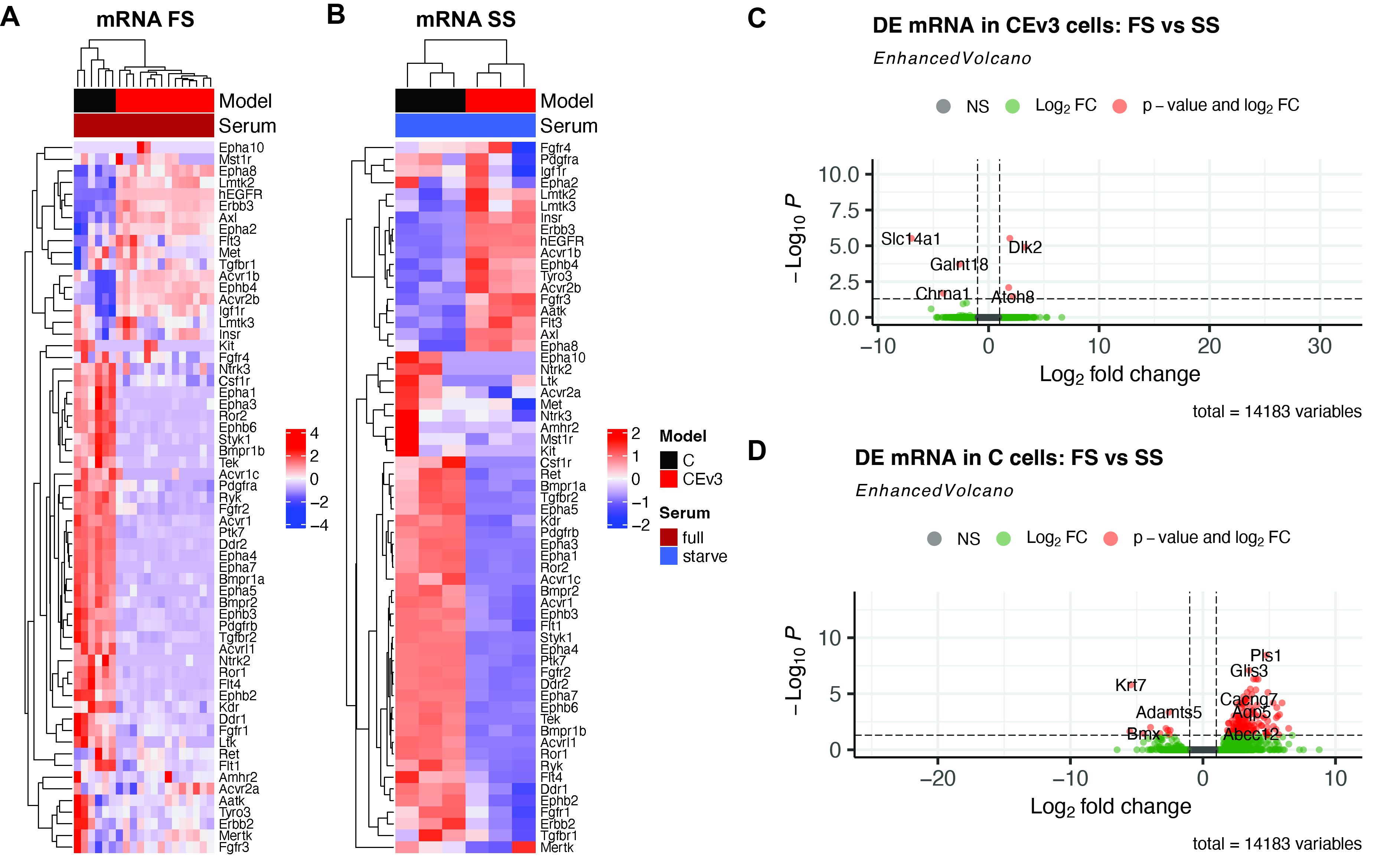

Supplement: Supplementary file 3 — Supplementary Material 3: Fig. S2 EGFRvIII (hEGFR) overexpression reprograms the receptor kinase transcriptome. Unsupervised hierarchical clustering heatmaps (AB) and volcano plots (CD) of DE receptor kinases at the mRNA level in both full (A) and starved (B) culture conditions show consistent downregulation in CEv3 (C) and C (D) cells. Full and starved serum cultures are denoted FS and SS, respectively [file 40478_2025_2068_MOESM3_ESM.tif]

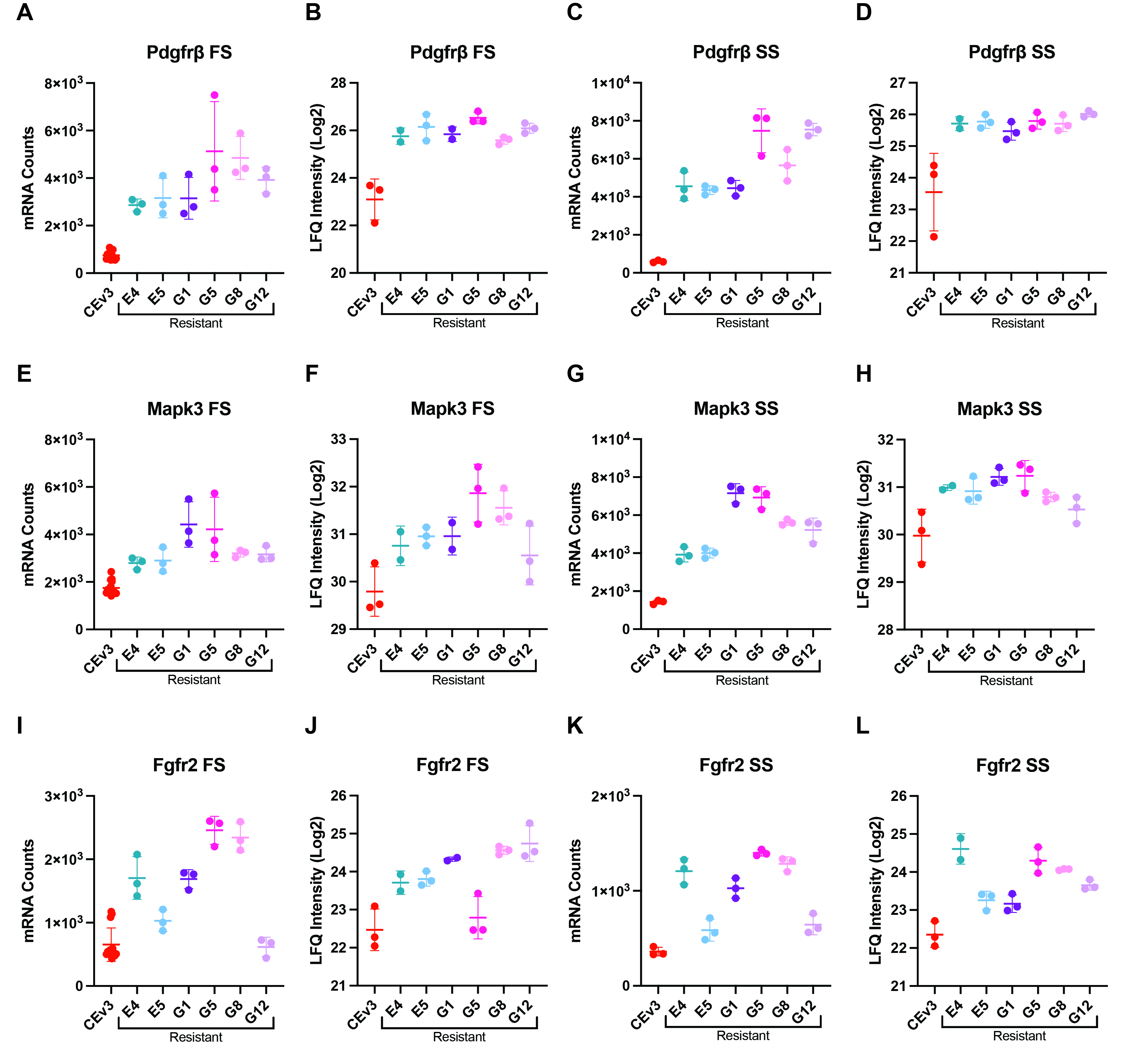

Supplement: Supplementary file 4 — Supplementary Material 4: Fig. S3 Acquired resistance alters specific kinases. mRNA (AC, EG, IK) and protein (BD, FH, JL) levels of Pdgfr (A-D), Mapk3 (E-H), and Fgfr2 (I-L) in resistant models compared to the parental CEv3 model [file 40478_2025_2068_MOESM4_ESM.tif]

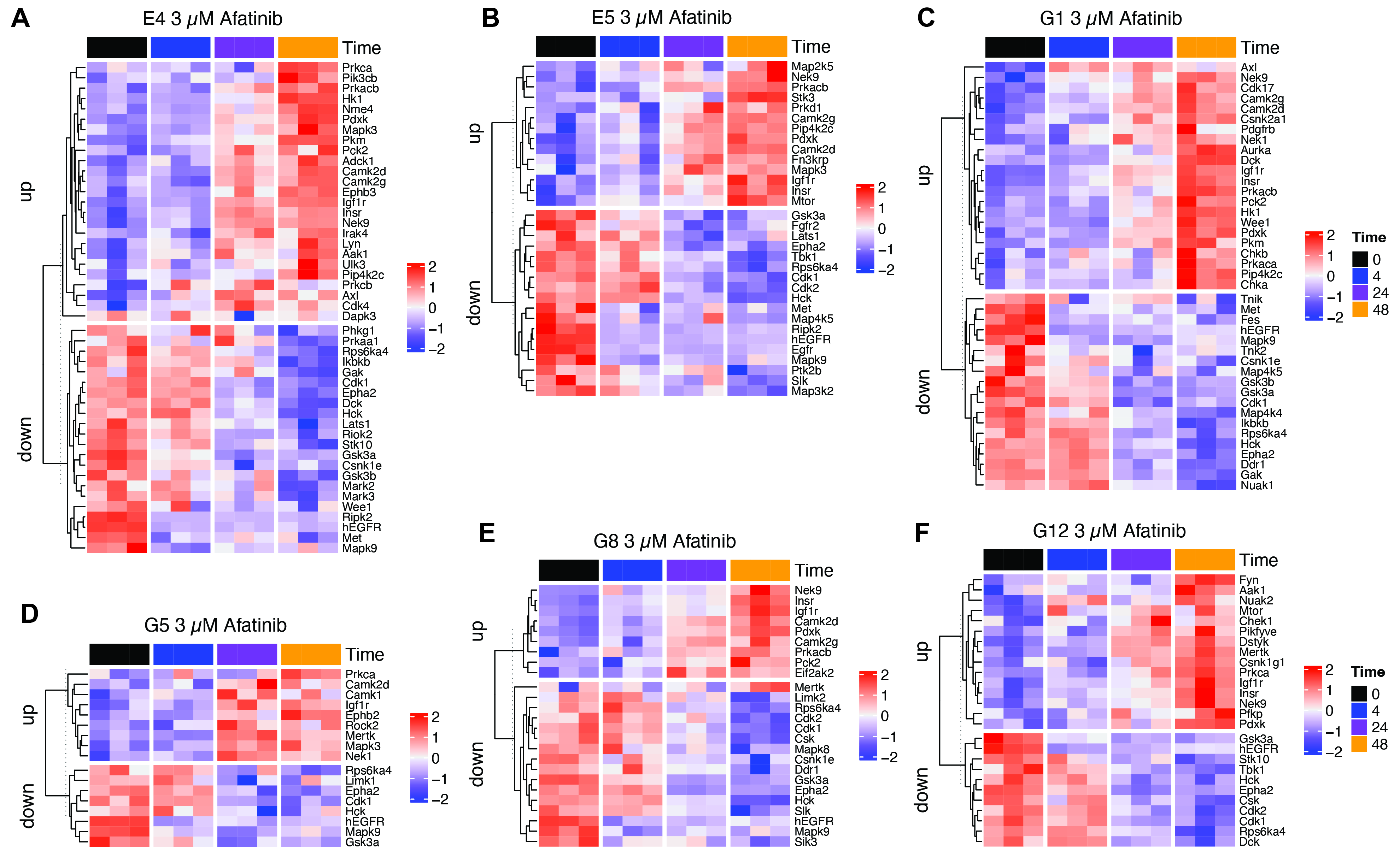

Supplement: Supplementary file 9 — Supplementary Material 9: Fig. S8 Afatinib induces acute kinome rewiring in resistant clones. K-means clustering identifies upregulated “up” or downregulated “down” DE kinase proteins after drug treatment. Heatmaps of DE kinases after drug treatment are shown for the following resistant lines compared to parental CEv3 cells: E4 (A), E5 (B), G1 (C), G5 (D), G8 (E), and G12 (F) [file 40478_2025_2068_MOESM9_ESM.tif]

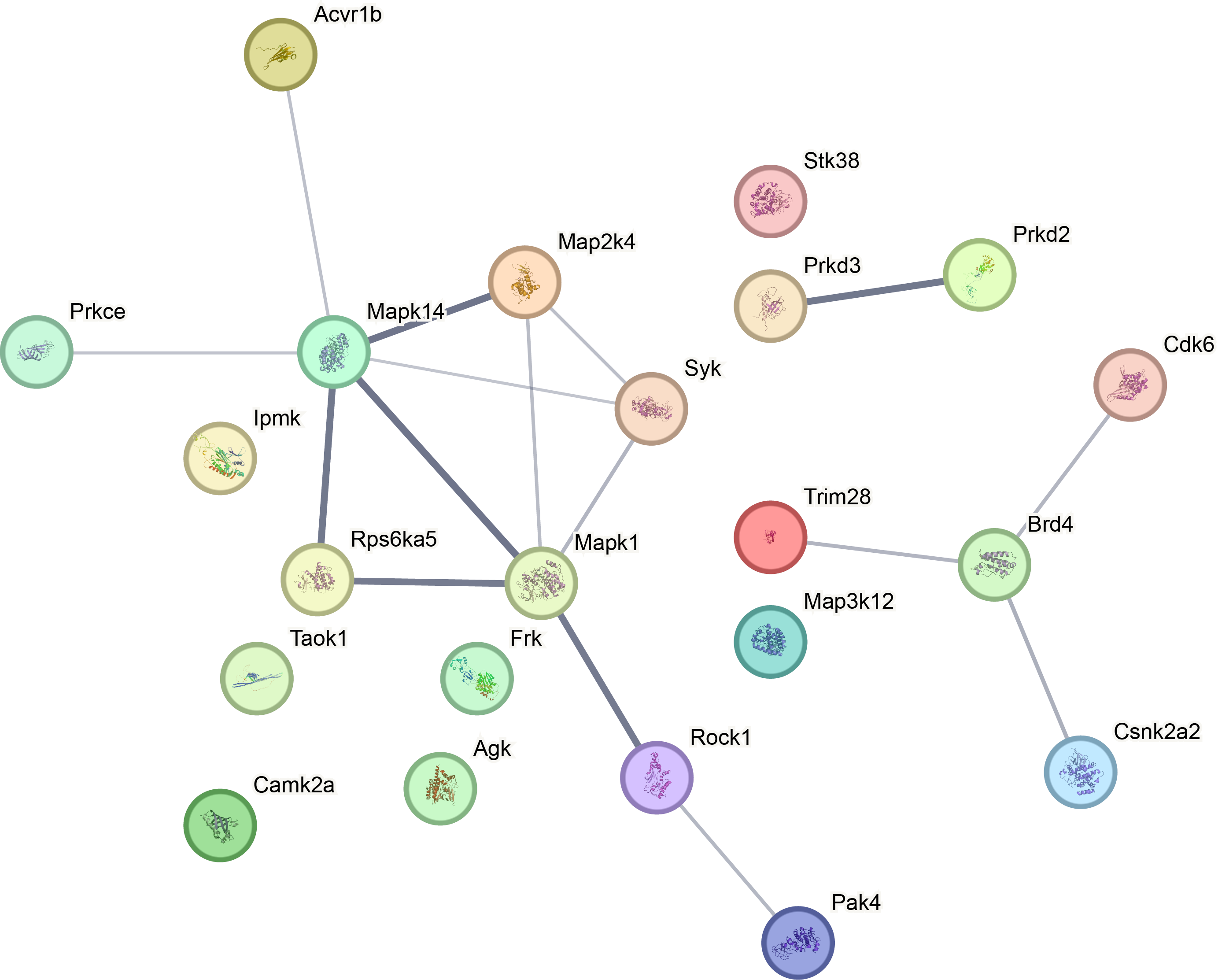

Supplement: Supplementary file 10 — Supplementary Material 10: Fig. S9 Interaction of upregulated DE Kinases associated with EGFR inhibition. Functional protein association network analysis using STRIND-DB (https://string-db.org/) shows interactions among upregulated kinases in the EGFRi signature [file 40478_2025_2068_MOESM10_ESM.tif]
